# The association between serum phosphorus and common carotid artery intima–media thickness in ischemic stroke patients

**DOI:** 10.3389/fneur.2023.1172488

**Published:** 2023-07-05

**Authors:** Huaping Du, Tingting Guo, Huan Ye, Yingshi Bao, Zhuoyin Qiu, Yaming Sun, Shoujiang You, Yuan Liu, Yuan Xu, Chunqing Zhang, Chunfang Qiu

**Affiliations:** ^1^Department of Neurology, Suzhou Ninth Hospital Affiliated to Soochow University, Suzhou, China; ^2^Department of Neurology, Zhangjiagang Traditional Chinese Medicine Hospital Affiliated to Nanjing University of Chinese Medicine, Suzhou, China; ^3^Department of Neurology, Suzhou Clinical Research Center of Neurological Disease, The Second Affiliated Hospital of Soochow University, Suzhou, China

**Keywords:** phosphorus, atherosclerosis, carotid artery intima–media thickness, acute ischemic stroke, predictor

## Abstract

**Purpose:**

An elevated concentration of phosphorus is associated with an increased risk of atherosclerosis and cardiovascular diseases. Common carotid artery intima–media thickness (cIMT) is an imaging marker of atherosclerosis. However, data on the relationship between phosphorus and cIMT in ischemic stroke are scarce. We aimed to evaluate the association between serum phosphorus levels and cIMT in patients who had experienced ischemic stroke.

**Patients and methods:**

A total of 1,450 ischemic stroke patients were enrolled. Participants were divided into four groups (quartiles) according to baseline serum phosphorus level. Carotid atherosclerosis was identified by measurement of cIMT; abnormal cIMT was defined as a maximum cIMT or mean cIMT ≥ 1 mm. Multivariable logistic regression models were used to assess the association between serum phosphorus level and the presence of abnormal cIMT.

**Results:**

In the multivariable adjusted analysis, falling into the highest quartile for serum phosphorus (Q4) was associated with a 2.00-fold increased risk of having abnormal maximum cIMT [adjusted odds ratio (OR) 2.00; 95% confidence interval (CI) 1.44–2.79] and a 1.76-fold increased risk of having abnormal mean cIMT (adjusted OR 1.76; 95% CI 1.22–2.53) in comparison to Q1. Furthermore, the association between serum phosphorus and abnormal cIMT was confirmed in analyses treating serum phosphorus as a continuous variable and in subgroup analyses.

**Conclusion:**

In acute ischemic stroke patients, baseline elevated serum phosphorus level was found to be independently associated with carotid atherosclerosis, as measured by cIMT.

## Introduction

Recent epidemiological data indicate that stroke remains the leading cause of death and disability in Chinese adults. Ischemic stroke, the most common type, accounts for 80% of all strokes. Atherosclerosis is a significant cause of ischemic stroke and is linked to an increased risk of recurrent stroke (1). Common carotid artery intima–media thickness (cIMT) is a recognized subclinical imaging marker of carotid atherosclerosis, with increased cIMT being associated with an elevated risk of cardiovascular disease (CVD), including stroke (2). Limited information is available on the determinants of atherosclerosis in Asian populations, and phosphorus may be an independent determinant; this was investigated in this study.

Serum phosphorus is widely distributed and plays a leading role in many biological processes, including cell signaling, bone metabolism, and muscle activity (3). Elevated phosphorus concentration is associated with an increased risk of CVD and all-cause mortality, as it triggers multiple pathophysiological processes, particularly in individuals with abnormal renal function (4, 5). Moreover, there is a positive association between higher serum phosphorus levels and cIMT, even among asymptomatic young adults (6, 7). Carotid atherosclerosis is a disease commonly associated with aging and is particularly prevalent among ischemic stroke patients. Additionally, an early study found that serum phosphorus levels increase with age (8). Given this evidence, we hypothesized that higher serum phosphorus levels would be associated with carotid artery atherosclerosis in acute ischemic stroke (AIS) patients. Hence, in the present study, we aimed to evaluate the association between serum phosphorus levels and cIMT in patients who had experienced ischemic stroke.

## Materials and methods

### Participants

We recruited patients who had experienced AIS or transient ischemic attack (TIA) from 22 hospitals in Suzhou between December of 2013 and May of 2014. A total of 3,720 patients aged ≥ 18 years with a clinical diagnosis of acute ischemic stroke or transient ischemic attack were considered eligible. Acute ischemic stroke was diagnosed by clinical data and computed tomography scan or magnetic resonance imaging of the brain according to the World Health Organization's criteria. Exclusion criteria were as follows: (1) final diagnosis of TIA; (2) time from onset to admission over 7 days; and (3) lack of carotid artery imaging data or serum phosphorus data. Following application of these criteria, a total of 1,450 patients with available data were included in the study (see Supplementary Figure 1 for a flowchart of participant selection).

### Data collection

Demographic and clinical data were collected via a standard questionnaire at admission; these included age, sex, lifestyle risk factors, vascular risk factors (hypertension, diabetes, atrial fibrillation, stroke, and coronary heart disease), and medication use. Information on these factors was also obtained through interviews with patients or members of their immediate family. Hypertension was defined as systolic blood pressure (BP) ≥ 140 mmHg and/or diastolic BP ≥ 90 mmHg, or use of antihypertensive medications. Diabetes mellitus was defined as fasting glucose ≥ 7.0 mmol/L (126 mg/dL), non-fasting glucose ≥ 11.1 mmol/L (200 mg/dL), with classic symptoms of hyperglycemia or hyperglycemic crisis and/or use of glucose-lowering drugs. Atrial fibrillation was defined as a history of atrial fibrillation, confirmed by ≥1 electrocardiograms or the presence of arrhythmia during hospitalization. The National Institutes of Health Stroke Scale (NIHSS), administered by trained neurologists, was used to evaluate stroke severity. Stroke was classified according to the Oxfordshire Community Stroke Project (OCSP) categorization by trained neurologists (9).

Blood samples were collected within 24 h of hospital admission. Serum phosphorus and as other laboratory variables were measured via assay at local laboratories.

### Carotid artery measurements

Carotid ultrasonography examinations were performed by certified sonographers who had received unified training and were unaware of the baseline characteristics and laboratory results of the participants (10). The region of interest for cIMT measurement was the far wall of the bilateral common carotid arteries, proximal to the bifurcation, along a plaque-free segment ≥ 10 mm long on the right and left sides (11). High-resolution B-mode ultrasound systems were used to measure cIMT. In each participating hospital, two sonographers with standardized training evaluated the carotid artery imaging data; discrepancies between their evaluations were resolved by consensus, and the final consistent determination of the two certified sonographers was recorded in each case.

The outcome variables were presence or absence of abnormality of maximum cIMT and abnormality of mean cIMT, defined as maximum cIMT ≥ 1 mm and mean cIMT ≥ 1 mm, respectively (11, 12). Maximum cIMT was defined as the larger of the measurements for the right and left common carotid arteries. Mean cIMT was calculated as the average of the measurements obtained for the right and left common carotid arteries.

### Statistical analysis

All analyses were conducted using the statistical software SPSS, version 17.0. Patients were categorized into four groups based on quartiles of serum phosphorus levels at admission: Q1 (<0.95 mmol/L), Q2 (0.95–1.08 mmol/L), Q3 (1.08–1.20 mmol/L), and Q4 (≥1.20 mmol/L).

Continuous variables are reported in the form mean ± standard deviation (SD) or median [interquartile range (IQR)] and were compared via analysis of variance or the Wilcoxon rank-sum test. Categorical variables are reported in the form frequency (%) and were compared using the chi-square test.

Multivariable logistic regression models were constructed to evaluate the association of serum phosphorus level with abnormal maximum cIMT and abnormal mean cIMT. Odds ratios (ORs) and 95% confidence interval (CIs) were calculated for each group, taking the group with the lowest serum phosphorus (Q1) as the reference. The covariates included in the multivariable models were selected based on prior knowledge and an association with *p* < 0.2 in the univariate analysis; these variables included age, sex, diastolic BP, current smoking status, alcohol consumption, history of hypertension, history of diabetes mellitus, history of atrial fibrillation, history of stroke, baseline NIHSS score, stroke syndrome, lipid-lowering medications, antiplatelet therapy, triglycerides (TG), total cholesterol (TC), low-density lipoprotein cholesterol (LDL-C), fasting plasma glucose (FPG), and estimated glomerular filtration rate (eGFR). To assess the robustness of the association between serum phosphorus level and abnormal cIMT, we also performed an analysis with serum phosphorus treated as a continuous variable. Subgroup analyses were conducted in the form of multivariate adjusted models stratified by age (≥70 years old vs. <70 years old), sex, cigarette smoking status, history of hypertension, and history of diabetes. All statistical tests were two-sided, and *P*-values < 0.05 were considered to represent statistical significance.

## Results

### Baseline characteristics

Data from a total of 1,450 acute ischemic stroke patients (872 men, 578 women) were analyzed. The mean age was 68.9 (12.2) years. The baseline characteristics of patients by serum phosphorus quartile are presented in Table 1. In comparison to participants with lower serum phosphorus levels, those with higher serum phosphorus levels were more likely to be younger, to be female, and to have experienced a longer delay from onset of symptoms to hospital admission; they also had a higher prevalence of prior diabetes mellitus, and were more likely to be receiving antihyperglycemic treatment. Patients with higher phosphorus levels also had higher levels of TG. Supplementary Table 1 shows the baseline characteristics of participants with and without abnormal maximum cIMT. Compared to those without abnormal maximum cIMT, patients with abnormal maximum cIMT tended to be older, were more likely to be male, and tended to have higher levels of LDL-C and phosphorus and a lower eGFR.

**Table 1:** Baseline characteristics of 1,450 acute ischemic stroke patients according to serum phosphorus quartile.

	**Serum phosphorus concentration, mmol/l**				
**Characteristics** ^*^	**Q1**<**0.95**	**Q2 0.95–1.08**	**Q3 1.08–1.20**	**Q4** ≥**1.20**	***P***
Number of subjects	355	351	330	414	
Demographics					
Age, y	70.9 ± 12.4	69.1 ± 11.9	68.5 ± 11.7	67.4 ± 12.4	0.001
Male sex	249 (70.1)	237 (67.5)	185 (56.1)	201 (48.6)	< 0.001
Cigarette smoking	76 (21.4)	71 (20.2)	77 (23.3)	88 (21.3)	0.799
Alcohol consumption	31 (8.7)	34 (9.7)	41 (12.4)	37 (8.9)	0.340
Clinical variables					
Time from onset to hospital, h	24.0 (5.0–48.0)	24.0 (6.0–48.0)	24.0 (7.0–72.0)	24.0 (7.0–72.0)	< 0.001
Baseline systolic BP, mmHg	153.3 ± 21.1	151.8 ± 21.7	152.0 ± 22.4	151.3 ± 22.5	0.652
Baseline diastolic BP, mmHg	85.6 ± 12.3	84.8 ± 12.3	84.9 ± 12.7	84.8 ± 12.6	0.803
TG, mmol/L	1.2 (0.8–1.6)	1.2 (0.9–1.8)	1.3 (0.9–1.7)	1.3 (1.0–2.0)	< 0.001
TC, mmol/L	4.7 (3.9–5.4)	4.6 (4.0–5.3)	4.6 (3.9–5.2)	4.8 (4.0–5.4)	0.226
LDL-C, mmol/L	2.8 (2.2–3.4)	2.7 (2.2–3.3)	2.7 (2.1–3.4)	2.8 (2.2–3.3)	0.853
HDL-C, mmol/L	1.2 (1.0–1.4)	1.2 (1.0–1.4)	1.2 (1.0–1.4)	1.2 (0.9–1.4)	0.296
FPG, mmol/L	5.9 (5.0–7.5)	5.5 (5.0–6.8)	5.7 (5.0–7.2)	5.7 (5.1–7.0)	0.266
eGFR, ml/min/1.73 m^2^	96.8 (79.9–117.4)	99.5 (80.4–120.0)	96.0 (78.2–120.1)	94.2 (74.7–117.0)	0.089
Baseline NIHSS score	4.0 (2.0–6.0)	3.0 (2.0–6.0)	3.0 (2.0–6.0)	3.0 (2.0–5.0)	0.381
Medical history					
History of hypertension	279 (78.6)	273 (77.8)	268 (81.2)	332 (80.2)	0.676
History of diabetes mellitus	75 (21.1)	81 (23.1)	98 (29.7)	123 (29.7)	0.010
History of coronary heart disease	23 (6.5)	17 (4.8)	15 (4.5)	15 (3.6)	0.325
History of atrial fibrillation	52 (14.6)	50 (14.2)	36 (10.9)	52 (12.6)	0.449
History of stroke	78 (22.0)	81 (23.1)	74 (22.4)	87 (21.0)	0.919
Medication history					
Antihypertensive therapy	209 (58.9)	202 (57.5)	193 (58.5)	249 (60.1)	0.908
Antiplatelet therapy	18 (5.1)	29 (8.3)	26 (7.9)	26 (6.3)	0.305
Statin therapy	10 (2.8)	9 (2.6)	11 (3.3)	15 (3.6)	0.834
Antihyperglycemic therapy	54 (15.2)	58 (16.5)	76 (23.0)	96 (23.2)	0.006
Thrombolysis treatment	8 (2.3)	2 (0.6)	9 (2.7)	6 (1.4)	0.139
Stroke syndrome					0.591
TACS	22 (6.2)	18 (5.1)	15 (4.5)	26 (6.3)	
PACS	193 (54.4)	191 (54.4)	177 (53.6)	208 (50.2)	
POCS	89 (25.1)	79 (22.5)	87 (26.4)	98 (23.7)	
LACS	51 (14.4)	63 (17.9)	51 (15.5)	82 (19.8)	

* Continuous variables are expressed in the form mean ± standard deviation or median (interquartile range). Categorical variables are expressed in the form frequency (percent).

BP, blood pressure; TG, triglycerides; TC, total cholesterol; LDL-C, low-density lipoprotein cholesterol; HDL-C, high-density lipoprotein cholesterol; FPG, fasting plasma glucose; eGFR, estimated glomerular filtration rate; NIHSS, National Institutes of Health Stroke Scale; TACS, total anterior circulation syndrome; PACS, partial anterior circulation syndrome; POCS, posterior circulation syndrome; LACS, lacunar syndrome; Q, quartile.

### Serum phosphorus and abnormal maximum cIMT

**Figure 1:**
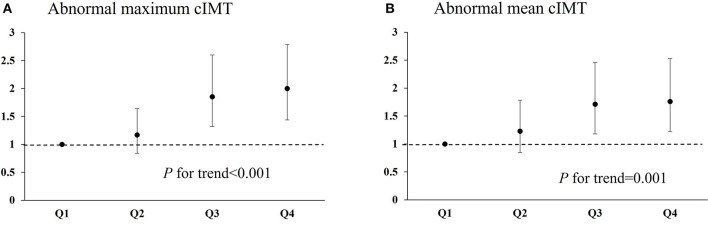
Relationship of serum phosphorus with abnormal maximum cIMT and abnormal mean cIMT in ischemic stroke patients. **(A)** Odds ratio for abnormal maximum cIMT among patients falling into each quartile for serum phosphorus level. **(B)** Odds ratio for abnormal mean cIMT among patients falling into each quartile for serum phosphorus level. cIMT, carotid artery intima–media thickness.

There were 538 patients (37.1%) with abnormal maximum cIMT. The relationship between serum phosphorus level and presence of abnormal maximum cIMT according to various models is shown in Table 2. In Model 1, adjusted for age and sex, the rate of abnormal maximum cIMT was significantly higher among participants who fell into the highest quartile in terms of phosphorus level at admission (≥1.20 mmol/L) compared with those who fell into the lowest quartile (<0.95 mmol/L) (OR 1.89; 95% CI 1.38–2.60). After further adjustments for TG, TC, LDL-C, NIHSS score, medical history, and other potential confounding factors in Model 3, the OR (95% CI) for abnormal maximum cIMT among patients falling into the highest quartile for phosphorus at admission as compared to those falling into the lowest quartile was 2.00 (95% CI 1.44–2.79) (Table 2; Figure 1A). Moreover, serum phosphorus was independently associated with increased odds of abnormal maximum cIMT after adjusting for possible confounding factors when serum phosphorus was treated as a continuous variable (OR 3.32; 95% CI 1.84–6.01) (Table 2).

**Table 2:** Odds ratios and 95% confidence intervals for abnormal maximum cIMT among acute ischemic stroke patients according to serum phosphorus level.

	**Serum phosphorus concentration by quartile, mmol/l**					**Serum phosphorus concentration as a continuous variable**	
	**Q1: < 0.95**	**Q2: 0.95–1.08**	**Q3: 1.08–1.20**	**Q4: ≥1.20**	***P* for trend**	**OR (95% CI)**	***P***
Median	0.86	1.01	1.13	1.30			
Cases, *n* (%)	118 (33.2%)	120 (34.2%)	135 (40.9%)	165 (39.9 %)			
Model 1	1.00	1.16 (0.84–1.60)	1.77 (1.28–2.46)	1.89 (1.38–2.60)	< 0.001	3.28 (1.83–5.88)	< 0.001
Model 2	1.00	1.16 (0.84–1.60)	1.74 (1.26–2.42)	1.86 (1.35–2.56)	< 0.001	3.17 (1.76–5.70)	< 0.001
Model 3	1.00	1.17 (0.84–1.64)	1.85 (1.32–2.60)	2.00 (1.44–2.79)	< 0.001	3.32 (1.84–6.01)	< 0.001

Model 1: adjusted for age and sex.

Model 2: further adjusted for current smoking status, alcohol consumption, history of hypertension, history of diabetes mellitus, history of atrial fibrillation, and history of stroke.

Model 3: further adjusted for NIHSS score at admission, DBP, stroke syndrome, use of lipid-lowering medications, antiplatelet therapy, FPG, TG, TC, LDL-C, and eGFR.

NIHSS, National Institutes of Health Stroke Scale; DBP, diastolic blood pressure; FPG, fasting plasma glucose; TG, triglycerides; TC, total cholesterol; LDL-C, low-density lipoprotein cholesterol; eGFR, estimated glomerular filtration rate.

### Serum phosphorus and abnormal mean cIMT

There were 418 patients (28.8%) with abnormal mean cIMT. The relationship between serum phosphorus level and presence of abnormal mean cIMT according to various models is presented in Table 3. In Model 1, adjusted for age and sex, the OR (95% CI) for abnormal mean cIMT among participants who fell into the highest quartile for serum phosphorus, compared with those who fell into the lowest quartile, was 1.79 (95% CI 1.27–2.51). Moreover, the relationship between falling into the highest quartile for serum phosphorus and having abnormal mean cIMT (OR 1.76; 95% CI 1.22–2.53) was still significant after further adjustment for TG, TC, LDL-C, NIHSS score, medical history, and other potential confounding factors in Model 3 (Table 3; Figure 1B). In addition, the association between serum phosphorus level and odds of abnormal mean cIMT remained significant (OR 2.37; 95% CI 1.24–4.51) when serum phosphorus was treated as a continuous variable (Table 3).

**Table 3:** Odds ratios and 95% confidence intervals for abnormal mean cIMT among acute ischemic stroke patients according to serum phosphorus level.

	**Serum phosphorus concentration by quartile, mmol/l**					**Serum phosphorus concentration as a continuous variable**	
	**Q1: < 0.95**	**Q2: 0.95–1.08**	**Q3: 1.08–1.20**	**Q4: ≥1.20**	***P* for trend**	**OR (95% CI)**	***P***
Median	0.86	1.01	1.13	1.30			
Cases, n (%)	92 (25.9%)	97 (27.6%)	104 (31.5%)	125 (30.2%)			
Model 1	1.00	1.23 (0.87–1.73)	1.69 (1.19–2.39)	1.79 (1.27–2.51)	< 0.001	2.68 (1.46–4.91)	0.001
Model 2	1.00	1.23 (0.87–1.73)	1.66 (1.17–2.36)	1.76 (1.25–2.47)	< 0.001	2.58 (1.40–4.74)	0.002
Model 3	1.00	1.23 (0.85–1.78)	1.71 (1.18–2.46)	1.76 (1.22–2.53)	0.001	2.37 (1.24–4.51)	0.009

Model 1: adjusted for age and sex.

Model 2: further adjusted for current smoking, alcohol consumption, history of hypertension, history of diabetes mellitus, history of atrial fibrillation, and history of stroke.

Model 3: further adjusted for NIHSS score at admission, DBP, stroke syndrome, use of lipid-lowering medications, antiplatelet therapy, FPG, TG, TC, LDL-C, and eGFR.

NIHSS, National Institutes of Health Stroke Scale; DBP, diastolic blood pressure; FPG, fasting plasma glucose; TG, triglycerides; TC, total cholesterol; LDL-C, low-density lipoprotein cholesterol; eGFR, estimated glomerular filtration rate.

### Subgroup analyses of serum phosphorus and abnormal maximum cIMT

Significant associations between admission serum phosphorus level and presence of abnormal maximum cIMT were observed in most subgroups (Table 4). However, no significant interactions were observed between phosphorus levels at admission and subgroup variables in terms of their association with presence of abnormal maximum cIMT (*P* for interaction > 0.05 in all cases).

**Table 4:** Odds ratios and 95% confidence intervals for abnormal maximum cIMT according to serum phosphorus quartile: subgroup analyses.

	**Serum phosphorus concentration by quartile**					
	**Q1**	**Q2**	**Q3**	**Q4**	***P* for trend**	***P* for interaction**
Across all participants	1.00	1.17 (0.84–1.64)	1.85 (1.32–2.60)	2.00 (1.44–2.79)	< 0.001	
Age, years						0.417
≥70 (median)	1.00	1.22 (0.79–1.89)	1.88 (1.20–2.95)	1.87 (1.20–2.91)	0.002	
< 70	1.00	1.12 (0.64–1.95)	1.96 (1.14–3.39)	2.36 (1.39–4.00)	< 0.001	
Sex						0.784
Female	1.00	1.68 (0.87–3.26)	2.52 (1.35–4.71)	2.63 (1.45–4.74)	0.001	
Male	1.00	1.04 (0.70–1.56)	1.66 (1.09–2.54)	1.93 (1.26–2.97)	0.001	
Cigarette smoking						0.144
No	1.00	1.01 (0.70–1.47)	1.72 (1.17–2.52)	1.72 (1.19–2.49)	0.001	
Yes	1.00	2.39 (1.03–5.52)	2.91 (1.29–6.55)	4.56 (2.03–10.22)	< 0.001	
History of hypertension						0.836
No	1.00	1.81 (0.82–3.99)	2.70 (1.16–6.27)	2.45 (1.12–5.35)	0.022	
Yes	1.00	1.07 (0.73–1.56)	1.73 (1.18–2.52)	1.98 (1.36–2.87)	< 0.001	
History of diabetes mellitus						0.336
No	1.00	1.45 (0.98–2.13)	2.20 (1.47–3.29)	2.40 (1.62–3.56)	< 0.001	
Yes	1.00	0.56 (0.27–1.17)	1.20 (0.61–2.35)	1.15 (0.59–2.24)	0.241	

Covariates were adjusted for the same variables as Model 3 in Table 2, with the exception of the stratified variable in each analysis.

## Discussion

In this multicenter population-based study, we have demonstrated that serum phosphorus levels at the time of hospital admission are associated with abnormal cIMT in patients with acute AIS. We found that the AIS patients with the highest levels of serum phosphorus appeared to have a 2.00-fold increased risk of abnormal maximum cIMT and a 1.76-fold increased risk of abnormal mean cIMT in comparison to those with the lowest serum phosphorus levels. Significant associations between serum phosphorus levels at admission and prevalence of abnormal cIMT were observed in most subgroups and in analyses in which serum phosphorus was treated as a continuous variable.

cIMT is a widely recognized marker of carotid atherosclerosis and has been shown to be a predictor of cardiovascular events, including stroke (12). A prespecified *post-hoc* analysis from the J-STARS (Japan Statin Treatment Against Recurrent Stroke) study indicated that participants in the highest cIMT quartile (≥0.931 mm) exhibited a higher incidence of atherothrombotic brain infarction compared to those in the lowest quartile (<0.812 mm) (13). A community-based prospective study from Japan also indicated that maximum cIMT was positively associated with risk of lacunar infarction, but not with non-lacunar infarction (14). In addition, a recent analysis as part of the CSPPT (China Stroke Primary Prevention Trial) revealed that elevated mean cIMT was positively correlated with risk of first ischemic stroke among individuals with hypertension, particularly among those with higher mean arterial pressure or diastolic blood pressure (15). A meta-analysis of 119 clinical trials involving 100,667 patients found that the extent to which cIMT progression was reduced by the effects of various interventions was predictive of degree of stroke risk reduction (16). Moreover, long-term treatment with statins has been shown to decrease cIMT, which may prevent recurrent stroke or cardiovascular events in patients who have experienced stroke (13, 17). However, early meta-analyses have not found conclusive evidence to support cIMT as an independent predictor of future cardiovascular events (18, 19). The variations observed among different studies in terms of their findings may be attributed to the lack of precision and reproducibility in early ultrasound examination techniques (20).

The available evidence indicates that an elevated phosphorus concentration is associated with abnormal cIMT. A recent study from South Korea indicated that serum phosphorus concentration is significantly associated with cIMT in asymptomatic menopausal women (21). In addition, a population-based cohort study of 13,340 participants without cardiovascular or renal disease demonstrated that serum phosphorus level is positively associated with cIMT in men (22). However, a study of 134 pediatric patients with chronic kidney disease found that phosphorus levels are negatively correlated with cIMT in this group (23). The disparity in findings could potentially be attributed to variations in the levels and distribution of levels of serum phosphorus across different age groups. In the present study of 1,450 AIS patients, 37.1% of whom had abnormal maximum cIMT, a significant association was observed between higher serum phosphorus level and presence of abnormal cIMT.

The exact mechanisms underlying the association between phosphorus and cIMT remain unclear. Several hypotheses have been proposed. First, thickening of the intima–media complex is associated with the calcification of vascular smooth muscle cells and the amount of extracellular matrix (24). Previous studies have indicated that elevated phosphorus levels are significantly associated with arterial calcification (25–27) and upregulation of the levels of matrix metalloproteinases (28, 29), which are reported to be associated with an increase in cIMT. Second, hyperphosphatemia may promote endothelial dysfunction, and reduced endothelial function has also been linked to an increase in cIMT (30, 31). Third, elevated phosphorus levels have also been found to be associated with increased oxidative stress, endoplasmic reticulum stress, and inflammatory response (32–34), all of which are known to be associated with elevated cIMT. Further studies are needed to elucidate the mechanism by which phosphorus promotes carotid intima–media thickening and atherosclerosis.

Our findings may have relevant implications for clinical practice. First, our results in AIS patients, together with the findings of a previous study in the general population (22), provide further evidence of the association between serum phosphorus and atherosclerosis. Second, our findings may help to explain the relationship between serum phosphorus and cardiovascular events through the pathogenesis of carotid atherosclerosis. Third, our data suggest that serum phosphorus could be a useful biomarker for carotid atherosclerosis that could be measured routinely as part of blood biochemistry investigations in AIS patients.

We recognize that this study has a number of limitations. First, with a cross-sectional study, it is difficult to demonstrate a causal link between phosphorus and cIMT, and we could not assess the prognostic value of serum phosphorus in terms of stroke recurrence due to a lack of follow-up data. Furthermore, phosphorus level is affected by various factors, such as thyroid disease and dietary intake, which also affect the occurrence of atherosclerosis. Unfortunately, we did not collect data on prior thyroid disease or dietary phosphorus intake. Additionally, some patients were excluded due to a lack of carotid artery imaging data or serum phosphorus data, which may have caused selection bias. Finally, we did not collect data on carotid artery stenosis; therefore, we were not able to further evaluate the association between serum phosphorus and carotid artery stenosis.

## Conclusion

Our results showed that an elevated serum phosphorus level at admission was positively correlated with cIMT among ischemic stroke patients, even after adjusting for traditional potential risk factors. Future prospective and randomization studies are needed to verify our findings in acute ischemic stroke patients as well as other populations.

## Data availability statement

The data that support the findings of this study are available on request from the corresponding author.

## Ethics statement

The studies involving human participants were reviewed and approved by the Ethics Committee of the Second Affiliated Hospital of Soochow University, as well as Ethical Committees at the participating hospitals. Written consent was obtained from all study participants or their immediate family members. The patients/participants provided their written informed consent to participate in this study.

## Author contributions

CQ, CZ, and YX contributed to conception and design of the study. YL and SY organized the database. SY performed the statistical analysis. HD wrote the first draft of the manuscript. TG, HY, YB, ZQ, and YS wrote sections of the manuscript. All authors contributed to manuscript revision, read, and approved the submitted version.
