# Supplementary material for: The association between serum phosphorus and common carotid artery intima–media thickness in ischemic stroke patients
Source: Front Neurol. 2023 Jul 5;14:1172488. doi: 10.3389/fneur.2023.1172488 (PMC10354419; doi:10.3389/fneur.2023.1172488)
Supplement: Supplementary file 1 [file Table_1.doc]

**Supplemental Table 1. Baseline characteristics of acute ischemic stroke patients**

**with normal and abnormal maximum cIMT**

| Characteristics ^a^ | Normal  cIMT | Abnormal cIMT | *P*-value |
| --- | --- | --- | --- |
|  |  |  |  |
| Number of subjects | 912 | 538 |  |
| Demographics |  |  |  |
| Age, y | 67.3±12.7 | 71.7±10.8 | <0.001 |
| Male sex | 512 (56.1) | 360 (66.9) | <0.001 |
| Cigarette smoking status | 188 (20.6) | 124 (23.0) | 0.276 |
| Alcohol consumption | 89 (9.8) | 54 (10.0) | 0.864 |
| Clinical features |  |  |  |
| Baseline systolic BP, mm Hg | 151.8±21.8 | 152.6±22.3 | 0.518 |
| Baseline diastolic BP, mm Hg | 85.4±12.3 | 84.3±12.8 | 0.096 |
| TG, mmol/L | 1.3 (0.9–1.8) | 1.2 (0.9–1.8) | 0.906 |
| TC, mmol/L | 4.6 (3.9–5.3) | 4.7 (4.0–5.4) | 0.066 |
| LDL-C, mmol/L | 2.7 (2.1–3.3) | 2.8 (2.2–3.5) | 0.044 |
| HDL-C, mmol/L | 1.2 (1.0–1.4) | 1.2 (1.0–1.4) | 0.085 |
| FPG, mmol/L | 5.7 (5.0–7.0) | 5.8 (5.1–7.4) | 0.067 |
| Phosphorus, mmol/L | 1.1 (0.9–1.2) | 1.1 (1.0–1.2) | 0.043 |
| eGFR, ml/min/1.73 m2 | 100.5 (81.1–121.4) | 90.8 (72.8–110.9) | <0.001 |
| Baseline NIHSS score | 3.0 (2.0–6.0) | 4.0 (2.0–6.0) | 0.496 |
| Medical history |  |  |  |
| History of hypertension | 715 (78.4) | 437 (81.2) | 0.198 |
| History of diabetes mellitus | 228 (25.0) | 149 (27.7) | 0.258 |
| History of coronary heart disease | 46 (5.0) | 24 (4.5) | 0.617 |
| History of atrial fibrillation | 112 (12.3) | 78 (14.5) | 0.227 |
| History of stroke | 197 (21.6) | 123 (22.9) | 0.576 |
| Medication history |  |  |  |
| Antihypertensive therapy | 531 (58.2) | 322 (59.9) | 0.543 |
| Antiplatelet therapy | 51 (5.6) | 48 (8.9) | 0.015 |
| Statin therapy | 28 (3.1) | 17 (3.2) | 0.924 |
| Antiglycemic therapy | 168 (18.4) | 116 (21.6) | 0.146 |
| Stroke syndrome |  |  | 0.918 |
| TACS | 49 (5.4) | 32 (5.9) |  |
| PACS | 480 (52.6) | 289 (53.7) |  |
| POCS | 225 (24.7) | 128 (23.8) |  |
| LACS | 158 (17.3) | 89 (16.5) |  |

^*^Continuous variables are expressed as mean ± standard deviation or as median (interquartile range). Categorical variables are expressed as frequency (percent).

**Abbreviations**: BP, blood pressure; TG, triglycerides; TC, total cholesterol; LDL-C, low-density lipoprotein cholesterol; HDL-C, high-density lipoprotein cholesterol; FPG, fasting plasma glucose; eGFR, estimated glomerular filtration rate; NIHSS, National Institutes of Health Stroke Scale; TACS, total anterior circulation syndrome; PACS, partial anterior circulation syndrome; POCS, posterior circulation syndrome; LACS, lacunar syndrome; Q, quartile.
