# Supplementary material for: The association between serum phosphorus and common carotid artery intima–media thickness in ischemic stroke patients
Source: Front Neurol. 2023 Jul 5;14:1172488. doi: 10.3389/fneur.2023.1172488 (PMC10354419; doi:10.3389/fneur.2023.1172488)
Supplement: Supplementary file 2 [file Presentation_1.pdf]

Figure legends

Supplemental Figure 1. Patient flowchart.

TIA indicates transient ischemic attack.
