# Supplementary figures and images for: The association between serum phosphorus and common carotid artery intima–media thickness in ischemic stroke patients
Source: Front Neurol. 2023 Jul 5;14:1172488. doi: 10.3389/fneur.2023.1172488 (PMC10354419; doi:10.3389/fneur.2023.1172488)

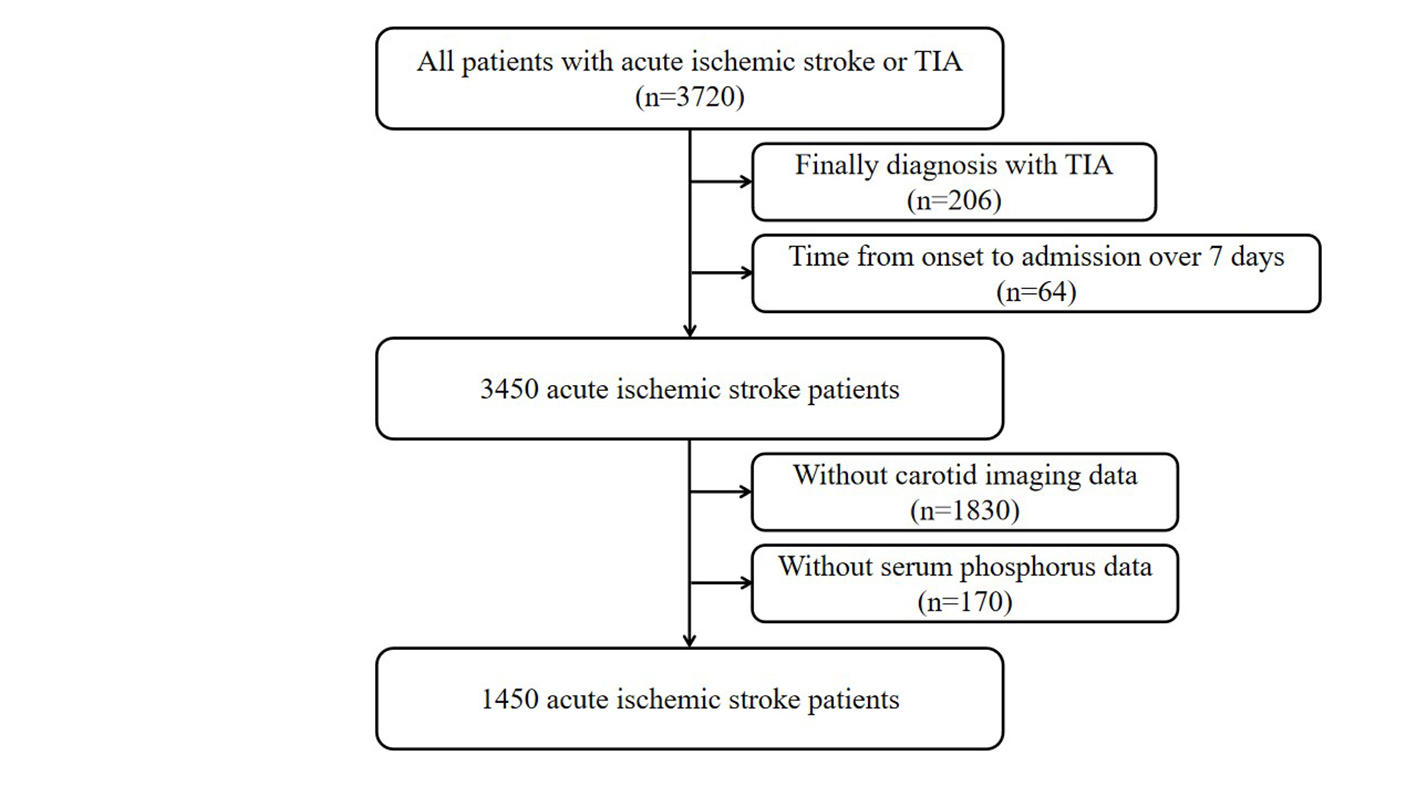

Supplement: Supplementary file 3 [file Image_1.jpg]
